# Reduction of methicillin-resistant *Staphylococcus aureus* biofilm growth and development using arctic berry extracts

**DOI:** 10.3389/fcimb.2023.1176755

**Published:** 2023-06-23

**Authors:** John Jairo Aguilera-Correa, Liisa Nohynek, Hanna-Leena Alakomi, Jaime Esteban, Kirsi-Marja Oksman-Caldentey, Riitta Puupponen-Pimiä, Teemu J. Kinnari, Ramon Perez-Tanoira

**Affiliations:** ^1^ Department of Clinical Microbiology, Instituto de Investigación Sanitaria (IIS)-Fundación Jiménez Díaz, Madrid, Spain; ^2^ Centro de Investigación Biomédica en Red de Enfermedades Infecciosas (CIBERINFEC), Instituto de Salud Carlos III, Madrid, Spain; ^3^ VTT Technical Research Centre of Finland Ltd., Industrial Biotechnology and Food, Espoo, Finland; ^4^ Department of Otorhinolaryngology – Head and Neck Surgery, Helsinki University Hospital, University of Helsinki, Helsinki, Finland; ^5^ Department of Clinical Microbiology, Hospital Universitario Príncipe de Asturias, Alcalá de Henares, Spain; ^6^ Department of Health Sciences, Faculty of Medicine, University of Alcalá, Alcalá de Henares, Spain

**Keywords:** methicillin-resistant *Staphylococcus aureus* (MRSA), biofilm, berry extract, surgical infection, antimicrobial

## Abstract

**Introduction:**

Surgical site infection remains a devastating and feared complication of surgery caused mainly by *Staphylococcus aureus* (*S. aureus*). More specifically, methicillin-resistant S. aureus (MRSA) infection poses a serious threat to global health. Therefore, developing new antibacterial agents to address drug resistance are urgently needed. Compounds derived from natural berries have shown a strong antimicrobial potential.

**Methods:**

This study aimed to evaluate the effect of various extracts from two arctic berries, cloudberry (*Rubus chamaemorus*) and raspberry (*Rubus idaeus*), on the development of an MRSA biofilm and as treatment on a mature MRSA biofilm. Furthermore, we evaluated the ability of two cloudberry seed-coat fractions, hydrothermal extract and ethanol extract, and the wet-milled hydrothermal extract of a raspberry press cake to inhibit and treat biofilm development in a wound-like medium. To do so, we used a model strain and two clinical strains isolated from infected patients.

**Results:**

All berry extracts prevented biofilm development of the three MRSA strains, except the raspberry press cake hydrothermal extract, which produced a diminished anti-staphylococcal effect.

**Discussion:**

The studied arctic berry extracts can be used as a treatment for a mature MRSA biofilm, however some limitations in their use exist.

## Introduction

Surgical site infection (SSI) is a devastating and feared complication of surgery. Before the antibiotic era the mortality rate from surgical infection remained extremely high. The risk for SSI remains important, especially in cases in which prosthetic-implanted materials are used. Such cases require antibiotic therapy, longer post-operative hospital stays, additional surgical procedures, treatment in intensive care units and can be associated with a higher mortality (Awad, 2012; Weiser et al., 2016). The global development of antibiotic resistance demands and requires us to find new solutions and methods to treat these infections such as the use of implants and sutures made of or coated with materials with antimicrobial properties (Leaper and Ousey, 2015). Moreover, the number of surgical procedures is increasing around the world (Weiser et al., 2016) and, accordingly, leads to a greater number of SSIs (Awad, 2012; Leaper and Ousey, 2015). In addition, incidence varies depending upon the type of procedure and the country in which it was performed (Saeed et al., 2015).

*Staphylococcus aureus (S. aureus)* is the most common microbe resulting in SSIs (Zarb et al., 2012). Among the isolated *S. aureus* SSI strains, a high percentage stem from methicillin-resistant *S. aureus* (MRSA) (Bhattacharya et al., 2016; Sganga et al., 2016). Interestingly, MRSA infections require up to a 5-day longer hospitalization compared with those caused by sensitive strains (Engemann et al., 2003), translating to higher healthcare costs (Rubin et al., 1999). SSI is considered a biofilm-related infection (Edmiston et al., 2015). Hence, any potential biofilm development of an infecting pathogen at the surgical site is necessary for the infection to establish itself, and, consequently, any anti-biofilm properties are essential to various treatment approaches.

The increasing MRSA resistance (Vestergaard et al., 2019) and reports of reduced susceptibility (Appelbaum, 2007) as well as complete resistance to glycopeptides (Szymanek-Majchrzak et al., 2018) all point towards the urgency in identifying alternative therapies for SSI. Plant extracts contain powerful molecules against this bacterium, e.g. *Phyllanthus emblica* and *Lycium shawii* (Tayel et al., 2018). As such, Nordic berry extracts, particularly those from the family Rosaceae, genus *Rubus* (Puupponen-Pimiä et al., 2005) and bilberry (*Vaccinium myrtillus* L.) (Burdulis et al., 2009), are interesting from this point of view. Previous studies have shown that antioxidants from arctic berry extracts such as ellagitannins have an antimicrobial effect on MRSA strains (Aguilera-Correa et al., 2021; Puupponen-Pimiä et al., 2021; Suriyaprom et al., 2022). Most of these studies have been conducted with collection strains showing less genetic variability than clinical strains isolated from infected patients and assessing antimicrobial capacity using a single methodology. Therefore, this study aimed to evaluate the effect of several berry extracts, namely, cloudberry (*Rubus chamaemorus* L.) and raspberry (*Rubus idaeus* L.), on MRSA biofilm development and as a potential treatment for a mature biofilm.

## Materials and methods

### Bacteria

We used three SSI-related MRSA strains isolated in the Department of Clinical Microbiology at the Fundación Jiménez Díaz University Hospital: the model strain (SAP 231) (Plaut et al., 2013) a strain of MRSA USA300 genetically modified to produce stably bioluminescence, and two clinical MRSA strains isolated from the infected wound of a 73-year-old male (MRSA1) and from the paronychia of a 92-year-old male (MRSA2), respectively. All strains were kept frozen at -80°C until the experiments were performed.

### Berry material

We evaluated a cloudberry seed-coat fraction [hydrothermal extract (CBSHE) and ethanol extract (CBSEE)], and a raspberry press cake wet-milled hydrothermal extract (RBHE).

The cloudberry material originated from Kiantama Oy (Suomussalmi, Finland) as a dry press cake consisting of the berry skin, flesh fractions and seeds as the major components. The press cake was stored at +15°C until processed, as described elsewhere (Aguilera-Correa et al., 2021; Puupponen-Pimiä et al., 2021).

Briefly, the skin and flesh fractions were removed using a vibrator sieve shaker, and the outermost layers of the cloudberry seeds were detached by sanding using an abrasive machine. The seed-coat powder obtained was stored frozen until used for the preparation of the hydrothermal and ethanol extracts.

For the raspberry, we used the press cake, a residue from juice and jam production. The press cake consisted primarily of the seeds and was stored frozen until ground. Briefly, the raspberry press cake was wet-milled twice using Masuko Sangyo’s MKZA10-15J Supermass collider. The grinding stone was a standard type MKGA10-46 made of aluminum oxide, with a rotation speed of 1500 rpm. The gap between the stones was 0.8 mm during the first pass and 0.3 mm during the second pass. The consistency of the berry material was 23.7%, resulting in a raspberry seed slurry, which was stored frozen until used for extraction.

### Extraction and refinement of the active berry components

#### Hydrothermal extraction

The hydrothermal extraction of the cloudberry seed-coat fraction (CBSHE) was previously described in detail by Puupponen-Pimiä et al (Suriyaprom et al., 2022). Briefly, for extraction, the dry seed-coat powder was mixed with water (1:20, w/v) and extracted for 1 h at 80°C. The suspension was filtered and extraction was repeated using the residue. The filtrates were pooled, freeze-dried and the extract was stored frozen.

The wet-milled raspberry slurry was liquid and, therefore, mixed with water at a ratio of 1:10 (w/v). The suspension was extracted for 1 h at 80°C and collected (RBHE) as described above for the cloudberry seed-coat fraction.

#### Ethanol extraction

For ethanol extraction, a dry cloudberry seed-coat fraction (CBSEE) was added to 80% EtOH (12:100, w/v). The ethanol was heated to 50°C before adding the cloudberry fraction, and the suspension was extracted for 1 min using a magnetic stirrer. The suspension was centrifuged for 15 min at 3000 rpm at room temperature, after which the extraction and separation of the phases were repeated twice using pellets. The supernatants were pooled and EtOH was evaporated in a Rotavapor at 35°C. The solid extract was removed from a Rotavapor using a few drops of water, frozen and then freeze-dried.

### Biofilm development studies

The biofilm development studies were carried out using the three above-mentioned strains. An overnight culture of each bacterium was grown in a tryptic soy agar (BioMérieux, France) at 37°C and 5% CO_2_. For each strain, 10^6^ colony-forming units (CFU)/ml was resuspended in saline (B. Braun, Germany). Next, 100 μL of this suspension were incubated at 37°C and 5% CO_2_ for 90 min in static conditions in a Nunc™ 96-well polypropylene MicroWell™ plate (Thermo Fisher Scientific, USA). After incubation, and medium removal, each well was washed twice with 100-μL of saline. Thereafter, in each well 150 µL of tryptic soy broth with 0.5% glucose as biofilm inductive growth medium was added (Aguilera-Correa et al., 2019), and inoculated with or without each extract (positive control) at different concentrations (32, 16 and 8 mg/mL, respectively), and incubated at 37°C and 5% CO_2_ for 24 h. Following incubation, each well was rinsed twice with 100-μL of saline, and, thereafter, 150-μL of tryptic soy broth with 10% of alamarBlue (BIO-RAD) (Pettit et al., 2005) was added and incubated at 37°C and 90 rpm for 30 min (Peeters et al., 2008). After incubation, the fluorescence was measured using an excitation wavelength of 560 nm and an emission wavelength of 590 nm. This experiment was performed in eight wells per extract concentration and completed in triplicate for each strain (*n* = 24).

#### Effect of berry extracts on preformed biofilm

To develop a mature biofilm, 10^6^ CFU/ml of each strain was resuspended in saline (B. Braun, Germany). Next, 100 μL of this suspension were incubated at 37°C and 5% CO_2_ for 90 min in static conditions in a Nunc™ 96-well polypropylene MicroWell™ plate (Thermo Fisher Scientific, USA). After incubation, each well was rinsed twice with 100-μL saline and 150-μL tryptic soy broth with 0.5% glucose with or without (positive control) each extract at different concentrations (32, 16 and 8 mg/mL, respectively) and, thereafter, incubated at 37°C and 5% CO_2_ for 24 h. Following incubation, each well was rinsed twice with 100-μL saline and 150-μL tryptic soy broth with 10% alamarBlue (BIO-RAD) (Pettit et al., 2005) and, then, incubated at 37°C and 90 rpm for 30 min (Peeters et al., 2008). After incubation, the fluorescence was measured using an excitation wavelength of 560 nm and an emission wavelength of 590 nm. This experiment was performed in eight wells per extract in triplicate for each strain (*n* = 24).

#### Effect of berry extracts on MRSA growth

The most active berry extract to inhibit biofilm development and to treat the mature MRSA biofilm from both previous experiments was used in this experiment. As such, 4 ml of Müeller-Hinton broth (Sigma Aldrich, United States) with or without (positive control) each extract was distributed in a well from a 12-well polypropylene plate (Thermo Fisher Scientific, USA) along with 200 µL of a 10^6^ CFU/ml from each MRSA strain in saline per well. These were then incubated at 37°C and 5% CO_2_ for 24 h. The bacterial viability was periodically measured at 1, 3, 6, 12 and 24 h, respectively, by taking a sample of 100 µL from each well and mixing it with 100-µL Müeller-Hinton broth supplemented with 20% of alamarBlue to reach a final concentration of 10% alamarBlue in a well from a Nunc™ 96-well polypropylene MicroWell™ plate. The plate was incubated at 37°C at 90 rpm for 30 min. Next, fluorescence was measured using an excitation wavelength of 560 nm and an emission wavelength of 590 nm following incubation. After the last measurement at 24 h, CFU per milliliter was estimated using the drop-plate method (Esteban et al., 2008) on mannitol salt agar plates. This experiment was performed in triplicate for each strain (*n* = 3).

#### The effect of berry extracts on biofilm development in a wound-like medium

The most effective concentration of the berry extract to inhibit biofilm development and to treat the mature MRSA biofilm from the previous experiments was used in this experiment. Biofilm development in a wound medium relies on a modification of the Lubbock chronic wound medium previously described (Sun et al., 2008; DeLeon et al., 2014). A wound-like medium is composed of 45% Bolton broth (Sigma-Aldrich, Spain), 50% bovine plasma (Sigma-Aldrich, Spain), 5% laked horse red blood cells (Fisher Scientific, United States), supplemented with one lyophilized BD BBL^™^ coagulase plasma and with or without (positive control) the berry extract concentration. Next, 1 ml of each kind of wound-like medium was distributed in a well from a 12-well polypropylene plate (Thermo Fisher Scientific, USA) along with 50 µL of a 10^8^ CFU/ml from each MRSA strain in saline per well and incubated at 37°C and 5% CO_2_ for 24 h. Following incubation, the content of each well was sonicated in a 50-mL CornStar™ conical tube (Corning Inc., United States) with 10-mL saline, using an Ultrasons-H 3000840 low-power bath sonicator (J. P. Selecta, Spain) at 22°C for 5 min (Esteban et al., 2008). This sonicated SS was serially diluted with saline and adhered CFU was estimated using the drop-plate method (Herigstad et al., 2001) on mannitol salt agar plates. This experiment was performed in triplicate for each strain (*n* = 3).

### Statistical analysis

All statistical analyses were performed using Stata Statistical Software, Release 11 (StataCorp, 2009). Data were evaluated using a one-sided Wilcoxon nonparametric test to compare two groups. We considered p ≤ 0.05 statistically significant. Results are presented as the median and interquartile range (IQR).

## Results

### The effect of berry extracts on biofilm development

**Figure 1:**
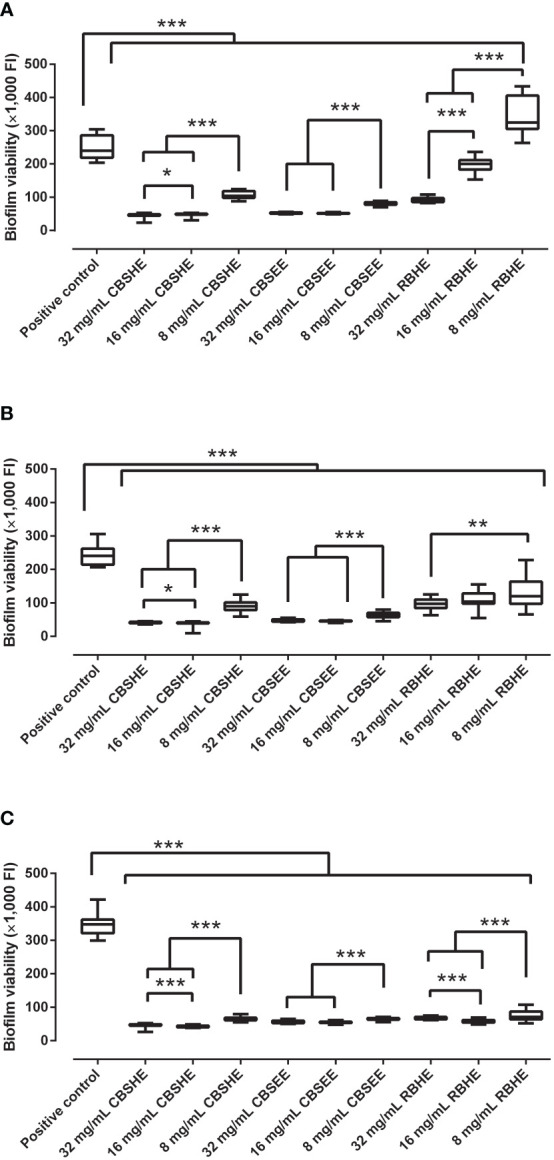
The effect of berry seed extracts on the biofilm development of SAP 231 **(A)**, MRSA1 **(B)** and MRSA2 **(C)**. FI: fluorescence intensity. *p < 0.05, **p < 0.01, ***p < 0.001 for the Wilcoxon test. After 24 h of biofilm development, each well was rinsed twice with saline, and, thereafter, tryptic soy broth with 10% of alamarBlue was added and incubated at 37°C and 90 rpm for 30 min. After incubation, the fluorescence was measured using an excitation wavelength of 560 nm and an emission wavelength of 590 nm. This experiment was performed in eight wells per extract concentration and completed in triplicate for each strain (*n* = 24).

CBSHE, CBSEE and RBHE showed a concentration-dependent inhibitory effect on MRSA biofilm development (**Figure 1**). Yet, for CBSEE, we detected no difference when comparing a concentration of 32 mg/mL or 16 mg/mL for any of the strains studied. All of the extracts produced a statistically significant reduction in the development of biofilms for the three MRSA strains, except RBHE which appeared to increase the development of SAP 231 biofilms by 35%.

At concentrations of 32 mg/mL and 16 mg/mL, CBSHE resulted in the largest reduction to the biofilm formation of the three MRSA strains, from 80.3–86.4% and 79.6–87.8%, respectively, compared with 78.3–83.7% and 78.4–84.1% for CBSEE and 59.6–80.2% and 16.8–83.4% for RBHE. Finally, at 8 mg/mL, CBSEE reduced the biofilm development for the three MRSA strains by 66–81.3%, CBSHE reduced development by 56.7–81.5% and RBHE by 50–80%.

### Biofilm treatment using berry extracts

**Figure 2:**
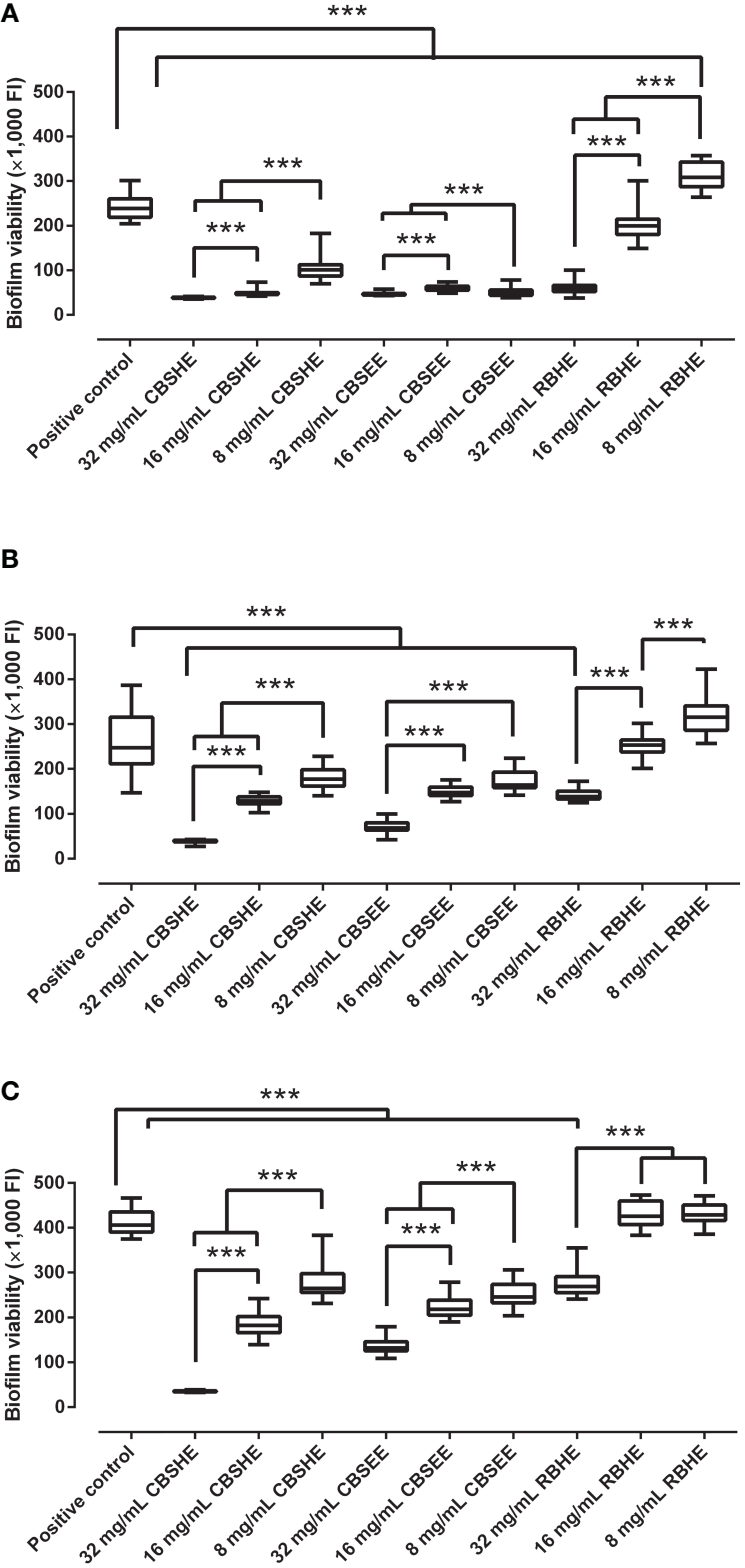
The effect of berry seed extracts on a 24-h biofilm growth of SAP 231 **(A)**, MRSA1 **(B)** and MRSA2 **(C)**. FI, fluorescence intensity. ***p < 0.001 for the Wilcoxon test. After 24 h of biofilm growth, each well was rinsed twice with saline, and, thereafter, tryptic soy broth with 10% of alamarBlue was added and incubated at 37°C and 90 rpm for 30 min. After incubation, the fluorescence was measured using an excitation wavelength of 560 nm and an emission wavelength of 590 nm. This experiment was performed in eight wells per extract concentration and completed in triplicate for each strain (*n* = 24).

Most of the berry extracts showed an inhibitory concentration-dependent effect as a treatment on a mature MRSA biofilm (**Figure 2**). CBSHE decreased mature biofilm growth of the three MRSA strains by 83.9–91.5% at 32 mg/mL, by 47.8–79.9% at 16 mg/mL and by 28.1–57.7% at 8 mg/mL when compared with the control. CBSEE decreased mature biofilm growth by 67.4–80.7% at 32 mg/mL, by 40.4–74.7% at 16 mg/mL and by 33.4–78.9% at 8 mg/mL. RBHE decreased the biofilm growth of SAP 231 by 33.7–74.9% at 32 mg/mL and by 16.5% at 8 mg/mL. However, at lower concentrations, RBHE increased the biofilm growth of MRSA1 and MRSA2 by 2.6–4.8% at 16 mg/mL and by 5.6–29.1 and 80%, respectively, at 8 mg/mL. Thus, RBHE was not included in further analyses.

### The effect of berry extracts on MRSA growth

**Figure 3:**
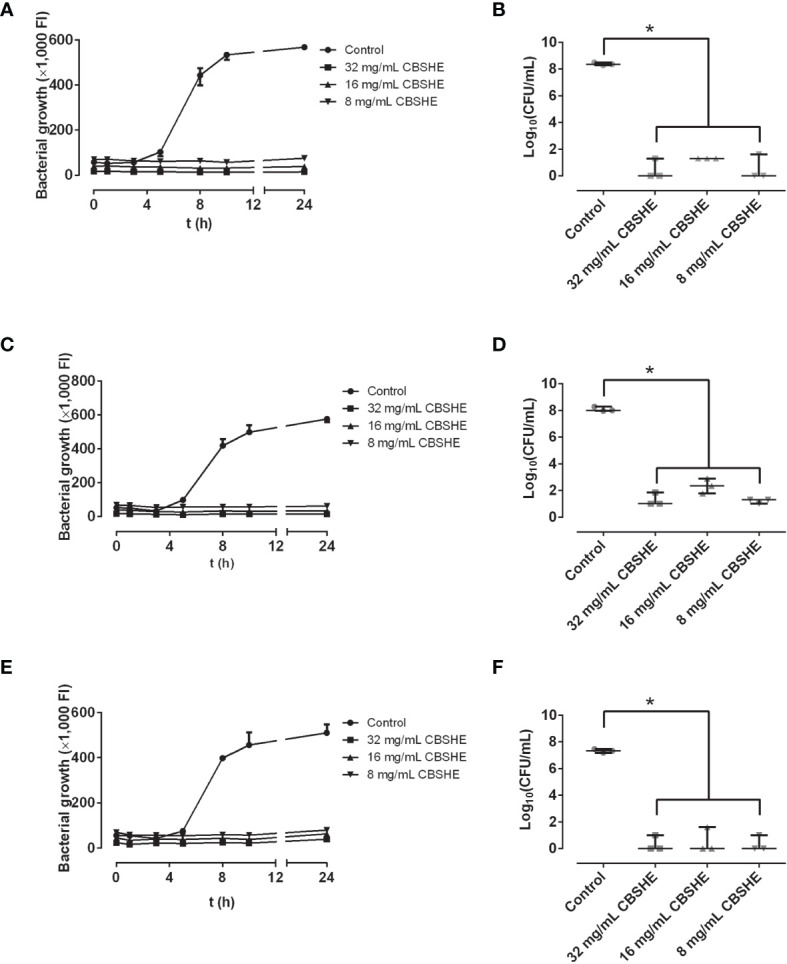
Growth curves (left column) and bacterial concentration (right column) of SAP 231 **(A, B)**, MRSA1 **(C, D)** and MRSA2 **(E, F)** in the presence of different concentrations of CBSHE. FI: fluorescence intensity. *p < 0.05 for the Wilcoxon test. Growth curves (left column) were performed by taking a sample of 100 µL of Müeller-Hinton broth with or without (positive control) each extract and mixing it with 100-µL Müeller-Hinton broth supplemented with 20% of alamarBlue, incubating at 37°C at 90 rpm for 30 min, and measuring the fluorescence at excitation/emission wavelength of 560/590 nm, respectively. Bacterial concentration (right column) was estimated after the last fluorescence measurement at 24 h, CFU per milliliter was estimated using the drop-plate method on mannitol salt agar plates. This experiment was performed in triplicate for each strain (*n* = 3).

CBSHE emerged as the most active of the berry extracts, showing a bactericidal concentration-dependent effect and significantly capable of decreasing the bacterial concentration. The best bactericidal concentration for all MRSA strains was found at 32 mg/mL (**Figure 3**).

### The effect of berry extracts on biofilm development in a wound-like medium

**Figure 4:**
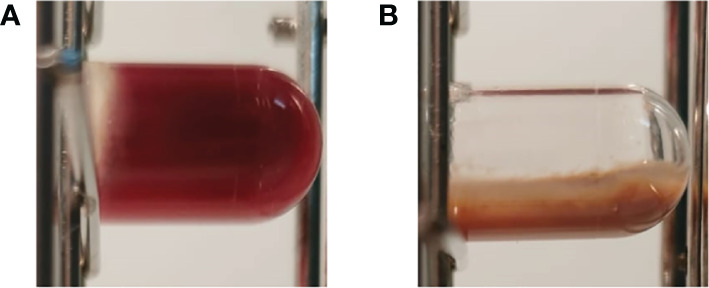
Macroscopic view of a wound-like medium with SAP 231 without **(A)** or with 32 mg/mL of CBSHE **(B)**.

**Figure 5:**
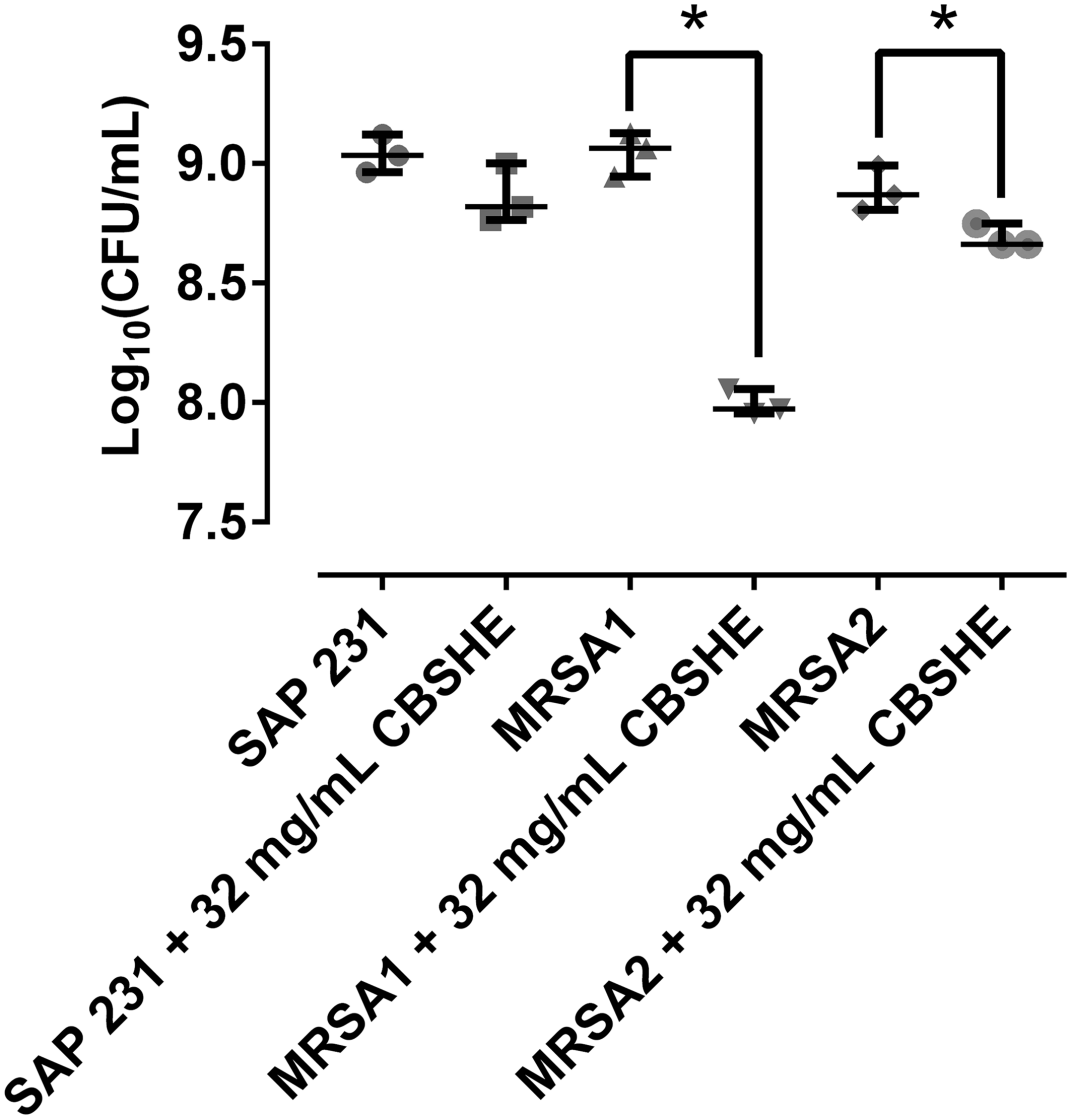
Bacterial concentration of each strain in a wound-like medium at 32 mg/mL of CBSHE. *p < 0.05 for the Wilcoxon test. The CFU per unit of volume were estimated from 1mL of each kind of wound-like medium with 50 µL of a 10^8^ CFU/ml from each MRSA strain incubated for 24 h, sonicated and quantified using the drop-plate method. This experiment was performed in triplicate for each strain (*n* = 3).

As shown in **Figure 4**, CBSHE macroscopically inhibited the coagulation of a wound-like medium compared with control samples in the three MRSA strains. The effects of CBSHE on biofilm development in a wound-like medium appear in **Figure 5**. As can be seen, at 32 mg/mL CBSHE significantly inhibited the biofilm development of MRSA1 and MRSA2 by 91.1% and 37.8%, respectively.

## Discussion

In this study, we report our findings on the ability of three berry extracts to prevent the biofilm development of methicillin-resistant *Staphylococcus aureus* (MRSA) using two different *in vitro* methods: biofilm development in tryptic-soy broth supplemented with 0.5% glucose and in a wound-like medium.

Based on our results, two cloudberry extracts (CBSHE and CBSEE) could prevent the biofilm development of the three MRSA strains we evaluated. The raspberry press cake hydrothermal extract provided a reduced anti-staphylococcal effect (**Figure 1**). Notably, we found that lower concentrations of this less active extract showed an unexpected opposite effect on the MRSA biofilm development, that is, 8 mg/mL of RBHE increased SAP 231 biofilm development by 35.4%. This finding is consistent with other reports, which assert that anti-staphylococcal compounds are found in raspberry blossoms (Pishgar and Makhfian, 2019). This antimicrobial ability may come from both the high phenolic concentrations and low concentrations of ascorbic acid which have been recently reported in the raspberry leaf buds (Krzepiłko et al., 2021). Furthermore, this phenomenon also indicates that it is necessary to also check the inter-strain effects, since each strain may show a dramatically different response to various concentrations and berry extracts (Nohynek et al., 2006). In addition, cloudberry seed extracts showed a high effect on MRSA biofilm development (**Figure 1**). This effect was concentration-dependent in all three MRSA strains since the highest concentration of the berry extract (32 mg/mL) associated with the highest inhibition of biofilm development (by 78.4–91.1%).

In our experiments, we tested approximately 1–3% berry extracts (8–32 mg/mL). These concentrations are significantly higher compared with antibiotic concentrations in systemic use, which fall in the mg/L range. This can be justified in several ways. First, antibiotics intended for topical use often consist of high concentrations (up to 3%), and we assume that berry extracts will be topical as well. The search, however, continues for individual active compounds isolated from these extracts, which could be as effective at a much lower concentration. Cloudberry contains citric and malic acids, α-tocopherol, anthocyanins and β-carotene. The main group of phenolic compounds in cloudberry are ellagitannins ∼3 g kg^−1^ (fw) and the content of anthocyanins is comparatively low (0.02 g kg^−1^, fw). The total ellagic acid content in dried leaves and fresh fruits of cloudberry is 70 g kg^−1^ and 0.6 g kg^−1^ respectively. The total phenolic content in fruits is 2.2–3 g kg^−1^ (fw), which can be assumed to represent mainly the ellagitannin content (Jaakkola et al., 2012). Finally, we are working with substances including phytochemicals like quercetin, chlorogenic acid and arbutin that may provide other benefits to humans, including their antioxidant attributes (Nohynek et al., 2006).

The effect of these berry extracts on treating a mature biofilm was slightly lower than their effect on biofilm development for the most anti-staphylococcal extracts. Analogous to our observation related to the inhibition of biofilm development, the cloudberry extract showed a higher anti-biofilm concentration-dependent effect than the raspberry hydrothermal extract. Once again, the latter showed an unexpected null or opposite effect on biofilm growth. This is due to one of the well-known inherent characteristics of bacterial biofilms, produced by extracellular polymeric substances, since this growth form confers resistance to nonspecific and specific host defenses during infection and confers a tolerance to an enormous plethora of antimicrobial agents (Flemming and Wingender, 2010).

Our results are in agreement with those of other researchers in the sense that cloudberry extracts can exhibit a bacteriocidic effect (Nohynek et al., 2006; Vučić et al., 2013) resulting from the antimicrobial activity of ellagitannins (Nohynek et al., 2006; Truchado et al., 2015), which may provoke the inhibition of extracellular microbial enzymes, depriving the substrates required for microbial growth (Silva et al., 2016; Díaz-Nuñez et al., 2021). This bacteriocidic effect may also reflect a direct action on microbial metabolism through an inhibition of oxidative phosphorylation or metal/iron deprivation (Scalbert, 1991; Nohynek et al., 2006).

Surgical site infections are often superficial, whereby the skin interacts with other damaged adjacent tissues and blood (Ki and Rotstein, 2008). As such, it is necessary to corroborate our anti-staphylococcal effect by using a medium with a composition closer to a real infection such as a wound-like medium (Peeters et al., 2008). This medium represents a realistic *in vitro* biofilm model simulating the functional characteristics of chronic pathogenic biofilms, allowing for the development of effective tools for treating such infections (Sun et al., 2008). Interestingly, the presence of the cloudberry seed extract prevented the coagulation of a wound-like medium. This may result from at least two related reasons. First, the extracts can interact with plasminogen and block its conversion from plasminogen to plasmin through staphylococcal coagulases (Cheng et al., 2010). This point is supported by research which concluded that only gallic acid–derived tannins can strongly interact with thrombin (Li et al., 2018). Second, the extracts at 32 mg/mL of CBSHE can exert a bacteriostatic or slightly bacteriocidic effect in a wound-like medium which reduces coagulase production in both situations.

## Conclusions

In summary, hydrothermal (CBSHE) and ethanol (CBSEE) extracts derived from arctic cloudberry (*Rubus chamaemorus*) seeds significantly inhibited MRSA biofilm formation and could be used as a treatment for a mature MRSA biofilm. Nevertheless, although they have a promising future, none of the extracts completely inhibited the development of a methicillin-resistant *Staphylococcus aureus* biofilm nor can they be used as a preventive treatment for surgical sites. Further studies are needed to elucidate the true molecular mechanisms of the observed inhibition and the effect of these extracts on cell growth.

## Data availability statement

The original contributions presented in the study are included in the article/supplementary material. Further inquiries can be directed to the corresponding authors.

## Author contributions

JJA-C, RP-P, RP-T and TJK contributed to the conception and design of the study. JJA-C created and managed the database. JJA-C performed the statistical analyses. JJA-C, RP-P, RP-T and TJK wrote the first draft of the manuscript. JJA-C, LN, H-LA, JE, K-MO-C, RPP, RP-T and TJK wrote sections of the manuscript. All authors contributed to revising, reviewing and approved the version of the manuscript submitted.
